# Ion Channels Orchestrate Pancreatic Ductal Adenocarcinoma Progression and Therapy

**DOI:** 10.3389/fphar.2020.586599

**Published:** 2021-01-19

**Authors:** Verena Hofschröer, Karolina Najder, Micol Rugi, Rayhana Bouazzi, Marco Cozzolino, Annarosa Arcangeli, Gyorgy Panyi, Albrecht Schwab

**Affiliations:** ^1^ Institute of Physiology II, University of Münster, Münster, Germany; ^2^ Department of Experimental and Clinical Medicine, Section of Internal Medicine, University of Florence, Florence, Italy; ^3^ Department of Biophysics and Cell Biology, Faculty of Medicine, University of Debrecen, Debrecen, Hungary

**Keywords:** pancreatic ductal adenocarcinoma, ion channels, therapy, immune cells, fibrosis

## Abstract

Pancreatic ductal adenocarcinoma is a devastating disease with a dismal prognosis. Therapeutic interventions are largely ineffective. A better understanding of the pathophysiology is required. Ion channels contribute substantially to the “hallmarks of cancer.” Their expression is dysregulated in cancer, and they are “misused” to drive cancer progression, but the underlying mechanisms are unclear. Ion channels are located in the cell membrane at the interface between the intracellular and extracellular space. They sense and modify the tumor microenvironment which in itself is a driver of PDAC aggressiveness. Ion channels detect, for example, locally altered proton and electrolyte concentrations or mechanical stimuli and transduce signals triggered by these microenvironmental cues through association with intracellular signaling cascades. While these concepts have been firmly established for other cancers, evidence has emerged only recently that ion channels are drivers of PDAC aggressiveness. Particularly, they appear to contribute to two of the characteristic PDAC features: the massive fibrosis of the tumor stroma (desmoplasia) and the efficient immune evasion. Our critical review of the literature clearly shows that there is still a remarkable lack of knowledge with respect to the contribution of ion channels to these two typical PDAC properties. Yet, we can draw parallels from ion channel research in other fibrotic and inflammatory diseases. Evidence is accumulating that pancreatic stellate cells express the same “profibrotic” ion channels. Similarly, it is at least in part known which major ion channels are expressed in those innate and adaptive immune cells that populate the PDAC microenvironment. We explore potential therapeutic avenues derived thereof. Since drugs targeting PDAC-relevant ion channels are already in clinical use, we propose to repurpose those in PDAC. The quest for ion channel targets is both motivated and complicated by the fact that some of the relevant channels, for example, K_Ca_3.1, are functionally expressed in the cancer, stroma, and immune cells. Only *in vivo* studies will reveal which arm of the balance we should put our weights on when developing channel-targeting PDAC therapies. The time is up to explore the efficacy of ion channel targeting in (transgenic) murine PDAC models before launching clinical trials with repurposed drugs.

## Introduction

Pancreatic ductal adenocarcinoma (PDAC) progresses rapidly once fully developed and can easily overcome current treatment strategies. The aggressiveness of the disease leads to a disastrous outcome for patients. The current 5-year survival rate is still less than 10% (Rawla et al., 2019). This poor prognosis is due to the absence of clinical symptoms in the early stage combined with the characteristic properties of PDAC: desmoplasia, early local invasion and metastasis, immune evasion, and resistance to radio- and chemotherapy. Desmoplasia involves a strong reaction of the tumor stroma in which matrix-producing pancreatic stellate cells (PSCs) play a central role. Mutual stimulation of tumor, stroma, and immune cells leads, in a positive feedback cycle, to enhanced growth factor and matrix production, creating a microenvironment conducive to tumor growth, migration/invasion of tumor cells into the tumor stroma, and metastasis (Mahadevan and Von Hoff, 2007; Sperb et al., 2020). Despite knowledge of many molecular details of PDAC, the therapeutic benefit derived thereof has been disappointingly small. Thus, there is an urgent need for novel concepts and therapeutic targets for the treatment of PDAC.

A promising novel concept is the targeting of ion channels in cancer. They are a major class of membrane proteins that have the ability to sense and modify properties of the tumor microenvironment and transduce signaling cascades triggered by its constituents. Therefore, they play central roles in signaling within and among tumor and stromal cells as well as in the coupling of extracellular events with cellular responses (Djamgoz et al., 2014). Ion channels are expressed in every cell where they exert cell-specific functions and housekeeping functions such as generating the membrane potential which, in turn, is a prerequisite for many processes such as Ca^2+^ signaling. Being the “working horses” of epithelial cells, ion channels are also essential for the normal function of the exocrine pancreas (e.g., Hayashi and Novak, 2013; Wang et al., 2013). Conversely, ion channel mutations cause hereditary diseases, so-called channelopathies. The most frequent one in Caucasians is the mutation of the cystic fibrosis transmembrane conductance regulator, CFTR, which causes, among others, a hereditary chronic pancreatitis which is a risk factor for developing PDAC (Becker et al., 2014).

Reviews from recent years on the role of ion channels in cancer (Djamgoz et al., 2014; Klumpp et al., 2018; Prevarskaya et al., 2018; Bulk et al., 2020; Ling and Kalthoff, 2020; Schnipper et al., 2020) predominantly focused on how ion channels shape the aggressive cancer cell behavior. However, cancer must be viewed as a complex tissue composed of different cell types. This is particularly relevant for PDAC: PSCs and immune cells are deeply involved in PDAC pathophysiology. PSCs have an important share in creating a tumor microenvironment in PDAC that contributes to immune evasion and thereby to the aggressiveness and therapy refractoriness of the disease (Wang et al., 2020b; Hessmann et al., 2020; Sperb et al., 2020). So far, there is still a considerable lack of knowledge on how ion channels and the (ionic) tumor microenvironment contribute to these aspects of PDAC pathophysiology. Yet, it has become evident that the function of ion channels in noncancerous cells has to be considered, too. Recent work in prostate cancer has started to go into this direction (Farfariello et al., 2020).

This review will put a special emphasis on the role of ion channels in stromal and immune cells. We will propose concepts on how fibrosis and immune evasion could be addressed in PDAC therapy by ion channel targeting. Due to their location in the plasma membrane, ion channels are easily accessible and well-characterized. Drugs targeting those have been in clinical use since decades. This is clearly exemplified by Na^+^ channel blockers acting as local anesthetics, antiarrhythmics, anticonvulsants, and diuretics. Moreover, there are drugs whose side effects involve ion channel blockade (e.g., K_V_11.1 blockade by haloperidol, fluoxetine, tamoxifen and amitriptyline (Pointer et al., 2017) or K_V_10.1 blockade by astemizole or imipramine (García-Ferreiro et al., 2004)). Other channel-targeting drugs such as the K_Ca_3.1 blocker senicapoc have gone through phase III clinical trials (Ataga et al., 2011). The availability of such drugs provides us with an enormous advantage as they may be repurposed within the cancer treatment context (Kale et al., 2015). While developing a new drug “from scratch” takes on average 12 years and costs one billion dollars, repurposing requires only 2–3 years and 10 million dollars (Zheng et al., 2013).

## Regulation of Pancreatic Cancer Cell Behavior by Ion Channels

### K^+^ Channels in Pancreatic Cancer Cells

By controlling the flow of potassium ions across the cell membrane, K^+^ channels regulate a multitude of processes, both in healthy and pathological conditions, including cancer (Huang and Jan, 2014). In proliferating cells, such as cancer cells, the K^+^ efflux mediated by K^+^ channels modulates cancer cell behavior by 1) providing the electrochemical force needed for the influx of Ca^2+^ (e.g., through store-operated Ca^2+^ channels (Feske et al., 2015)), which is known to be important for G_0_/G_1_ and G_1_/S transitions; 2) by transiently hyperpolarizing the membrane potential, which is also an important feature for cell cycle progression (Urrego et al., 2014); or by 3) being involved in cell volume regulation that highly relies on K^+^ efflux (Hoffmann et al., 2009). K^+^ channels may also work in a nonconductive manner by promoting signal transduction pathways involved in cell proliferation through interaction with other membrane proteins such as integrins (see below) (Becchetti et al., 2019).

Voltage-gated K^+^ channels (K_V_ channels) are a large family of 40 genes grouped into 12 subfamilies. One of their key functions is the repolarization of the cell membrane potential of excitable cells (Wulff et al., 2009; Arcangeli and Becchetti, 2017). However, they are also found in nonexcitable cells where K_V_ channels play important roles in cell proliferation, Ca^2+^ signaling, migration, and cell volume regulation. Moreover, they promote cancer progression (Huang and Jan, 2014). The involvement of ion channels in PDAC, such as K_V_ channels, is summarized in Table 1.

**TABLE 1:** Ion channel expression and their functional role in pancreatic cancer and stellate cells.

Channel	Function	Reference
**K^+^ channels**		
K_Ca_3.1	Functional expression in PDAC cell lines and elevated expression in PDAC tissue; cell proliferation	Jäger et al. (2004)
	Functional expression in PDAC cells; cell migration, proliferation, and invasion	Bonito et al. (2016)
	Subset of PDAC cell lines: Oxygen consumption, ATP production, and cellular proliferation	Kovalenko et al. (2016)
	Expression in PSC; migration and chemotaxis, [Ca^2+^]_i_ signaling, calpain activity, functional cooperation with TRPC3	Storck et al. (2017)
	High expression correlates with poor patient prognosis in PDAC	Zaccagnino et al. (2016); Jiang et al. (2017)
	Macrophage infiltration into cancer tissue via a Ca^2+^-dependent activation of CXCL5-CCL20 secretion by PDAC cells	Jiang et al. (2019)
K_ir_3.1	Highly expressed in PDAC	Brevet et al. (2009).
K_2P_1.1 (TWIK-1)	mRNA up-regulation in PDAC tissue	Williams et al. (2013).
K_2P_2.1 (TREK-1)	Expressed in PDAC cells (BxPC-3)	Sauter et al. (2016).
	Mediates pH-sensitive K^+^ current	
	Modulates the membrane potential (V_m_)	
	PSCs: mRNA expression	Fels et al. (2016)
K_2P_3.1 (TASK-1)	mRNA down-regulation in tissues from PDAC patients	Williams et al. (2013)
K_V_1.3	Reduced primary tumor weight *in vivo* by inhibitor clofazimine	Zaccagnino et al. (2017)
	Decreased expression in PDAC, associated with metastatic tumors	Brevet et al. (2009)
*Mitochondrial channel* mitoK_V_1.3	Apoptosis of cancer cells, cancer development, and progression in mouse models of PDAC	Leanza et al. (2017) and Zaccagnino et al. (2017)
K_V_10.1 (hEAG)	Inhibition of channel activity by monoclonal antibodies; inhibition of tumor cell growth in mouse xenograft model of pancreatic cancer	Gómez-Varela et al. (2007)
K_V_11.1 (hERG)	Expression in PDAC samples	Zhou et al. (2012)
	Cell growth and invasiveness	Feng et al. (2014)
	PDAC malignancy *in vitro* and *in vivo*; diagnostic and prognostic biomarker	Lastraioli et al. (2015)
	PDAC cell migration, modulator of f-actin organization, and Ca^2+^ signaling	Manoli et al. (2019)
**TRP channels**		
TRPC1	TGF-β stimulated Ca^2+^-responses; migration and invasion (BxPc3 cells)	Dong et al. (2010)
	Mechanosignaling of murine PSC, pressure-dependent PSC activation	Fels et al. (2016)
TRPC3	Up-regulated in PDAC stroma; functional cooperation with K_Ca_3.1; PSC migration and chemotaxis; and Ca^2+^ signaling	Storck et al. (2017)
TRPC6	PSCs: Cell migration, Ca^2+^ signaling, and cytokine secretion in hypoxia	Nielsen et al. (2017)
TRPM2	SIRT6-elevated ADPr levels increase TRPM2 activation; migration (BxPc3 cells)	Bauer et al. (2012) and Lin et al. (2018)
TRPM7	Overexpressed in PDAC tissue; correlated with poor patient survival	Rybarczyk et al. (2012)
	Overexpression correlates with increased tumor size and advanced tumor stages	Yee et al. (2015)
	PDAC cell invasion in Panc-1/MiaPaCa2; expression in lymph node metastasis and primary tumor correlation in human PDAC	Rybarczyk et al. (2017)
TRPM8	Up-regulated in PDAC cell lines and tissue; cell proliferation	Yee et al. (2010)
	Functional expression in the plasma membrane; cell migration (Panc-1 cells)	Cucu et al. (2014)
TRPV1	Overexpressed in PDAC and the involved neurons; potential link to pain intensity reported by cancer patients	Hartel et al. (2006)
TRPV4	Prolonged high fat/alcohol exposure increases TRPV4 expression in PSCs, fibrosis	Zhang et al. (2013)
	Pressure-modulated mRNA expression in PSCs	Fels et al. (2016)
TRPV6	Up-regulated in pancreatic cancer tissue; affects proliferation, migration, invasion, and apoptosis in PDAC	Song et al. (2018)
	Down-regulated in PDAC cell line and in the tumor epithelium of PDAC tissue	Zaccagnino et al. (2016) and Tawfik et al. (2020)
	Loss of function variants linked to early onset chronic pancreatitis (a risk factor for PDAC development)	Masamune et al. (2020)
**Other ion channels**	
**ASICs**
ASIC1, ASIC3	Functional (over-)expression in PDAC; [Ca^2+^]_i_ signaling, EMT, liver and lung metastasis	Zhu et al. (2017)
**P2X receptors**		
P2X7	PSC proliferation and death	Haanes et al. (2012)
	Overexpressed in PDAC cell lines; cell survival, migration, and invasion	Giannuzzo et al. (2015)
	Tumor growth; PSC number/activity, fibrosis	Giannuzzo et al. (2016)
**Piezo1**	High mRNA levels in PSCs	Fels et al. (2016)
	PSCs: Ca^2+^ influx, cytoskeletal architecture, cell invasion, pH-dependent mechanosensation	Kuntze et al. (2020)
**ORAI1/STIM1**	Prosurvival antiapoptotic role by mediating store-operated Ca^2+^ entry	Kondratska et al. (2014)
**CaCC** (TMEM16A)	Functionally overexpressed in human PDAC cells; supports migration, but not proliferation	Sauter et al. (2015)
	Promotes pathogenesis of acute pancreatitis via IP_3_R/Ca^2+^/NFκB/IL-6 signaling	Wang et al. (2020a)
	Essential for EGF-induced store-operated Ca^2+^ entry during pancreatic cancer cell migration; overexpression correlates with low patient survival probability	Crottès et al. (2019)

**K**_**V**_ **1.3 channels:** In healthy humans, the K_V_1.3 channels are mainly expressed in the central nervous system and in immune cells (Cahalan and Chandy, 2009). K_V_1.3 channel expression is found up-regulated in several PDAC cell lines (Zaccagnino et al., 2017). Overexpression of the channel is an advantage for cancer cells to promote proliferation and cell survival. This was illustrated by targeting PDAC cells with a K_V_1.3 inhibitor (clofazimine) which induces apoptosis and reduces the weight of tumors grown from orthotopically transplanted PDAC cells (Zaccagnino et al., 2017). K_V_1.3 is also expressed in the mitochondria (mitoK_V_1.3), where it regulates apoptosis in PDAC cells (Leanza et al., 2017). The above-cited data from PDAC cell lines, however, differ from those obtained in tissue samples from PDAC patients where K_V_1.3 expression is down-regulated. This down-regulation correlates with metastasis. The diminished expression of K_V_1.3 was explained as a result of the methylation of its promoter (Brevet et al., 2009).

**K**_**V**_ **10.1 and K**_**V**_ **11.1 channels:** The EAG family of voltage-gated K^+^ channels comprises at least two members, K_V_10.1 (EAG1) and K_V_11.1 (hERG1), which are deeply involved in the regulation of different cancer hallmarks (Pardo and Stühmer, 2014). These channels have been identified as a potential target for anticancer therapies (Arcangeli and Becchetti, 2017; Xu et al., 2018), and both channels are expressed in PDAC (K_V_10.1 (Gómez-Varela et al., 2007); K_V_11.1 (Feng et al., 2014; Lastraioli et al., 2015)).

In the healthy organism, K_V_10.1 and K_V_11.1 are expressed in excitable cells such as neurons and muscle cells. In addition to its expression in PDAC, both channels have been detected in many other tumor cell lines and primary tumors including neuroblastoma (Meyer and Heinemann, 1998; Pardo et al., 1999), melanoma (Nilius and Wohlrab, 1992; Meyer et al., 1999; Gavrilova-Ruch et al., 2002) as well as different tumors of epithelial origin (Ouadid-Ahidouch et al., 2001; Lastraioli et al., 2004; Hemmerlein et al., 2006; Ding et al., 2007; Ousingsawat et al., 2007), and leukemias (Pillozzi et al., 2002). The expression of K_V_10.1 seems to correlate with high-grade tumors and may confer a proliferative advantage for tumor cells (Comes et al., 2015).

K_V_11.1 channels are preferentially expressed in cardiac myocytes and required for the ordered repolarization of cardiac action potentials. K_V_11.1 expression in cancer cells has also been linked to high-grade tumors and has been strongly implicated in cell proliferation and migration of several cancers (Comes et al., 2015). K_V_11.1 expression is elevated in PDAC tumor cells, in particular in lymph node–positive PDAC (Feng et al., 2014). In contrast, cells of the tumor stroma and the normal ductal epithelium do not express K_V_11.1 (Lastraioli et al., 2015). K_V_11.1 supports cancer cell proliferation, vitality, migration, and invasion also in several PDAC cell lines (J. Feng et al., 2014; E. Lastraioli et al., 2015; Zhi et al., 2017). It is involved in cell cycle regulation as K_V_11.1 silencing promotes cell cycle arrest in the G_1_ phase (Feng et al., 2014). In primary PDAC cultures, K_V_11.1 blockage was found to be cytotoxic.

K_V_11.1 physically and functionally interacts with other plasma membrane proteins, such as the epidermal growth factor receptor (EGF-R) and adhesion receptors of the integrin family (Arcangeli and Becchetti, 2006; Lastraioli et al., 2015), which strongly contribute to PDAC aggressiveness (Sun et al., 2018). In addition, EGF-R inhibition represents one of the therapeutic strategies for nonresectable PDAC (Hessmann et al., 2020). The interaction between K_V_11.1 and EGF-R stimulates an EGF-R–dependent phosphorylation of ERK1 and ERK2, which are key signaling proteins downstream to EGF-R, and are involved in cell proliferation (Lastraioli et al., 2015). As stated in the introduction about K^+^ channels, K_V_11.1 modulates cell proliferation through a conductive mechanism by its impact on the cell membrane potential (Becchetti et al., 2017). K_V_11.1 also regulates cell migratory programs of PDAC cells by modulating stress fiber dynamics and f-actin organization by its impact on the intracellular Ca^2+^ concentration (Manoli et al., 2019). This effect relies on nonconductive mechanisms and is based on the formation of a complex with β1 integrins, which leads to the activation of downstream signaling processes involving paxillin.

K_V_11.1 is a target for both posttranscriptional and posttranslational modifications by small noncoding RNA molecules (miRNAs). miRNAs participate in human tumorigenesis and/or metastasis because of their ability to target oncogenes and/or tumor suppressors (Feng et al., 2014). K_V_11.1 is a direct target of mir-96 and mir-493 in human PDAC (Feng et al., 2014; Zhi et al., 2017; Xu et al., 2018), where both miRNAs are down-regulated. These data are recapitulated in PDAC cell lines. *In vivo* and *in vitro*, mir-96 and mir-493 silencing increases proliferation, migration, and invasion of PDAC cells, while their overexpression highly suppresses tumorigenicity and metastasis of PDAC. These observations suggest that the above miRNAs can work as tumor suppressors in PDAC in a K_V_11.1-dependent manner (Feng et al., 2014; Zhi et al., 2017).

**K**_**Ca**_ **3.1 channels:** K_Ca_3.1 channels are functionally expressed in pancreatic ducts and are part of the transepithelial ion and fluid transport machinery (Hayashi et al., 2012; Wang et al., 2013). K_Ca_3.1 channels are found in the luminal and basolateral membranes in the intercalated and interlobular ducts of the pancreas (Hayashi and Novak, 2013).

K_Ca_3.1 is one of the first K^+^ channels that were found to be massively overexpressed in primary pancreatic cancer samples and to be functional in several pancreatic cancer cell lines (Jäger et al., 2004). Such findings were later reproduced by other groups (Zaccagnino et al., 2016; Jiang et al., 2017, 2019). K_Ca_3.1 expression rises in a stepwise fashion during the dedifferentitation process from the normal pancreas to PanINs and PDAC (Jiang et al., 2017). The clinical relevance of this finding is underscored by the correlation of increased K_Ca_3.1 channel expression and patient prognosis: high K_Ca_3.1 channel expression is associated with poor patient survival. The predictive power of K_Ca_3.1 expression is not limited to PDAC. It also applies to several other cancer entities including, among others, breast (Faouzi et al., 2016), lung (Bulk et al., 2015), and ovarian cancer (Zhao et al., 2016).

Mechanistically, K_Ca_3.1 channels regulate pancreatic cancer cell behavior in several ways. First of all, they provide the electrochemical driving force needed for Ca^2+^ entry by counterbalancing the depolarization of the membrane potential caused by Ca^2+^ influx channels such as TRP channels or Cl^−^ efflux through anion channels. The former has been observed in PSCs (Storck et al., 2017) and is known from many immune cells such as macrophages (Gao et al., 2010) and lymphocytes (see *Ion Channel Involvement in Desmoplasia*). The latter appears to be relevant for pancreatic cancer cells. K_Ca_3.1 channels interact with the gamma-aminobutyric acid (GABA) receptor subunit pi (GABRP). Thereby, they maintain the cell membrane potential and allow efficient Ca^2+^ signaling to enhance CXCL5-CCL20 secretion. This, in turn, causes macrophage infiltration into the cancer tissue and tumor growth (Jiang et al., 2019). K_Ca_3.1-mediated K^+^ efflux is also necessary for volume dynamics during the cell cycle (Bonito et al., 2016) and migration. Accordingly, K_Ca_3.1 can promote tumor progression by modulating cell proliferation as well as cell migration and invasion (Schwab et al., 2012; Bonito et al., 2016). Finally, K_Ca_3.1 channels are not only expressed in the plasma membrane but also in the inner membrane of mitochondria (De Marchi et al., 2009). There is indirect evidence that K_Ca_3.1 channels are also present in the mitochondria of pancreatic cancer cells and regulate metabolic activity of mitochondria, potentially by modulating their membrane potential (Kovalenko et al., 2016). However, the relative importance of the plasma membrane vs. mitochondrial K_Ca_3.1 channels in regulating the cellular metabolism still remains to be determined. The common link could be the intracellular Ca^2+^ concentration, which also affects mitochondrial function (Delierneux et al., 2020).

**K**_**2P**_ **channels:** There is very limited information about K_2P_ channels in pancreatic cancer. A systematic review of public databases identified the up- or down-regulation of K_2P_1.1 or K_2P_3.1 mRNA, respectively. However, these findings were not complemented by any functional data (Williams et al., 2013). K_2P_2.1 modulates migration and proliferation of PDAC cell lines (Sauter et al., 2016).

### TRP Channels in Pancreatic Cancer Cells

**TRPM channels:** An analysis of published genomic data from PDAC patients revealed an overexpression and the occurrence of somatic mutations of TRPM2. Both of them are negatively correlated with patient survival. TRPM2 overexpression or silencing modulates migration and proliferation of a PDAC cell line. So far, it remains to be determined how the somatic mutations of TRPM2 affect channel activity (Lin et al., 2018).

Similar observations were made for TRPM7. It is overexpressed in PDAC tissue, and this correlates with poor patient survival (Rybarczyk et al., 2012; Rybarczyk et al., 2017) as well as increased tumor size and advanced PDAC stages (Yee et al., 2015). In the zebra fish model, TRPM7 contributes to the development of the pancreas and carcinogenesis (Yee et al., 2011). Somatic TRPM7 mutations have been detected in several cancer entities (reviewed in Yee (2017)). Their functional significance has yet to be determined. On the cellular level, TRPM7 regulates proliferation and cell cycle progression (Yee et al., 2011). In zebra fish, the defects in cell cycle progression of the *trpm7* ^b508^ mutants can be partially rescued by supplementary Mg^2+^ (Yee et al., 2011). TRPM7 knockdown reduces PDAC cell chemotaxis and invasion (Yee et al., 2015), at least in part by regulating the intracellular Mg^2+^ homeostasis and via the Hsp90α/uPA/MMP-2 proteolytic axis (Rybarczyk et al., 2012, Rybarczyk et al., 2017).

TRPM8 is also overexpressed in human PDAC compared to normal tissue and required for cell proliferation (Yee et al., 2010). PDAC cells express functional TRPM8 channels as shown by whole-cell patch-clamp experiments. Channel activation inhibits PDAC cell motility (Cucu et al., 2014). Moreover, TRPM8 silencing increases the sensitivity to gemcitabine (Liu et al., 2018).

**TRPV6:** The high Ca^2+^ selectivity is a distinguishing feature of TRPV6 (and TRPV5) channels (Fecher-Trost et al., 2017). So far, there are only very few publications on TRPV6 channels in pancreas physiology and pathophysiology. Immunohistochemistry revealed their expression in acinar cells (Zhuang et al., 2002). A transcriptomic analysis indicates however that they are expressed at higher levels in the ductal epithelium (Segerstolpe et al., 2016).

Overexpression of TRPV6 appears to be common in cancers of epithelial origin. Thus, its tumor-promoting role in prostate cancer is well established (Raphaël et al., 2014). However, there is a controversy with respect to TRPV6 expression in PDAC. While Song et al. (2018) reported an overexpression, we found a reduced expression in microdissected PDAC samples (Zaccagnino et al., 2016). However, both of these studies did not take into account whether the tissue samples were from invasive or noninvasive parts of the tumor. This is a relevant distinction: A preponderance of TRPV6 expression was shown for the invasive parts of breast cancer (Dhennin-Duthille et al., 2011). Loss-of-function variants of TRPV6 channels are linked to another pancreas pathology: Early onset chronic pancreatitis (Masamune et al., 2020). We already mentioned in the introduction that an early onset (hereditary) chronic pancreatitis, which can also be caused by a mutation of the CFTR channel, leads to an increased risk to develop PDAC (Becker et al., 2014). The potential clinical relevance of TRPV6 channels in PDAC is further underpinned by observations from a phase I dose escalation study with the TRPV6 inhibitor SOR-C13 in cancer patients. Stable disease and a reduction in the CA 19-9 tumor biomarker were observed in both PDAC patients participating in this study (Fu et al., 2017).

### Cl^−^ Channels in Pancreatic Cancer Cells

ANO1 (TMEM16A) is a Ca^2+^-activated Cl^−^ channel (CaCC). In freshly isolated murine pancreatic acini, HCO_3_^−^ exits the cells through the apical ANO1 channel, which controls luminal pH balance. Luminal pH may be perturbed by the exocytotic release of the acid content of zymogen granules, both under physiologic condition and upon supramaximal stimulation, which represents an *in vitro* model of acute pancreatitis (Han et al., 2016). In acute pancreatitis, IL-6 promotes ANO1 expression via IL-6R/STAT3 signaling. ANO1 overexpression, in turn, increases IL-6 secretion via IP_3_R/Ca^2+^/NFκB signaling activation (Wang et al., 2020a). Thus, ANO1 appears to be involved in a positive feedback loop in this inflammatory disorder.

CFTR and ANO1 are highly expressed in Capan-1 cells, where they mediate ATP/UTP-regulated Cl^−^ secretion (Wang et al., 2013). ANO1 is overexpressed in several PDAC cell lines when its expression is compared to that in HDPE cells which are suggested to represent a model of the normal human pancreatic ductal epithelium (Sauter et al., 2015). Indeed, the analysis of patient material shows that ANO1 mRNA and protein are up-regulated in 75% of the cases. This is associated with a poor probability of survival (Crottès et al., 2019).

An EGFR-related signaling pathway, requiring ANO1, regulates Cl^−^ and Ca^2+^ homeostasis in pancreatic cancer cells. This EGF-induced store-operated Ca^2+^ entry is required for the migration of pancreatic cancer cells (Crottès et al., 2019). Interestingly, ANO1 has a promigratory role in PDAC cells but has no effect on cell proliferation. Whole-cell patch-clamp experiments reveal functional ANO1 as a major mediator of PDAC CaCC currents. While knockdown of ANO1 using siRNA nearly completely abolishes the CaCC-mediated currents, the three tested ANO1 inhibitors T16Ainh-A01, CaCCinh-A01, and NS3728 show unspecific side effects and limited specificity (Sauter et al., 2015).

## Ion Channel Involvement in Desmoplasia

**FIGURE 1:**
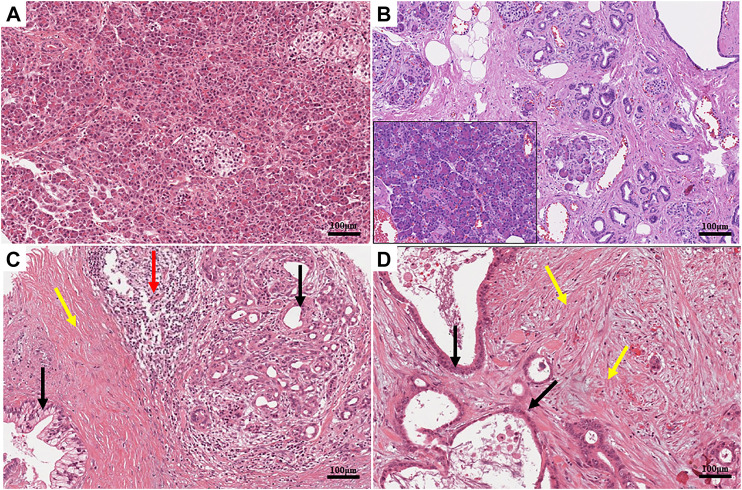
**(A)** Histomorphology of a healthy human pancreas, hematoxylin and eosin (H&E). The parenchymal structure of the organ is clearly visible. Acinar cells are identifiable by their typical round shape. Their bases are stained in blue due to the presence of the nuclei, while their apices are pink due to the high concentration of zymogen. Two islets are located in the central and right parts of the image. The cytoplasm of the islet cells is paler than the surrounding acinar cells. **(B)** Histomorphology of a chronic pancreatitis, hematoxylin and eosin (H&E). The tissue is characterized by an evident increase in interlobular fibrosis, atrophy of the acini, and inflammatory infiltrate, which is evident when compared to the healthy component of the same sample (inset). **(C**,**D)** Histomorphology of two human pancreatic ductal adenocarcinomas (PDACs), hematoxylin and eosin (H&E). The normal architecture of the parenchyma is lost. Multiple layers of cells highlight the neoplastic lesions in panel **(C)**. High levels of desmoplasia (colored in pink) are present especially in panel **(D)**. Distribution of different cell populations is detectable in the tumor tissue; neoplastic cells (pointed by black arrows) are embedded in a dense desmoplastic stroma (pointed by yellow arrows). Evident immune cells infiltration (pointed by red arrows) is present on the right side of the figure. Immune cells are identifiable by their small sizes and the intense basophilic staining of the nuclei. Scale bars: 100 μm.

Fibrosis is a pathological outcome common for many chronic inflammatory diseases including chronic pancreatitis (Wynn and Ramalingam, 2012). The abundant stroma reaction (desmoplasia) is a hallmark common to both chronic pancreatitis and PDAC (Haeberle et al., 2018). Chronic pancreatitis is considered a risk factor for pancreatic cancer, and indeed, it frequently evolves to a true PDAC (McKay et al., 2008). In both cases, the normal pancreatic parenchyma is markedly remodeled (as shown in Figure 1) so that the normal organ function is eventually lost. The poorly vascularized desmoplastic tissue is characterized by high stiffness, low elasticity, and high tissue pressure (up to 100 mmHg) (Stylianopoulos et al., 2012; Fels et al., 2016; Pethő et al., 2019), which leads to impaired perfusion of the tumor tissue with the further result of tissue hypoxia. The pancreatic stellate cells (PSCs) are believed to be the key effectors behind the stroma deposition in PDAC and chronic pancreatitis (Haeberle et al., 2018). Desmoplasia represents an important challenge that new PDAC therapies have to deal with (Henke et al., 2020). The absence of vascularization combined with vessel compression because of the massive fibrosis prevents the efficient delivery of the chemotherapeutic drugs (Dauer et al., 2018).

Consequently, new strategies targeting the stroma compartment have emerged. This includes the attempt to attenuate/reverse the activation of the cancer-associated fibroblasts (CAFs) which also includes PSCs. The results of these studies however are contradictory. Inhibiting the TGF-β signaling pathway with the anticancer compound Minnelide, which is able to reverse the activation state of the CAFs, has a similar positive effect in a murine PDAC model (Dauer et al., 2018) as the inhibition of hedgehog signaling in CAFs with IPI-926. Moreover, IPI-926 also increases the delivery and the efficacy of gemcitabine in mice (Olive et al., 2011). However, other studies highlighted that an uncontrolled depletion of the stroma compartment rather promotes PDAC progression than slowing it down (Özdemir et al., 2014). Consequently, the understanding of the stromal compartment in PDAC has to be further refined. It has become apparent that cancer-associated fibroblasts constitute a heterogeneous cell population with distinct gene expression profiles, location within the tumor, and function (Von Ahrens et al., 2017; Öhlund et al., 2017; Miyai et al., 2020). Öhlund et al. propose a distinction between inflammatory fibroblasts, mainly responsible for the secretion of inflammatory factors, and myofibroblasts that are responsible for the ECM production (Öhlund et al., 2017). PSCs are included in this last category. To the best of our knowledge, it has not yet been studied whether these two types of CAFs are also equipped with distinct sets of ion channels.

### Pancreatic Stellate Cells

In a healthy pancreas, PSCs are usually kept in a quiescent state, and they are responsible for the maintenance of the tissue integrity by regulating the ECM turnover (Haeberle et al., 2018). In PDAC, PSCs become strongly activated by the secretome and the physicochemical properties of the PDAC microenvironment (Omary et al., 2007a). Thus, PSCs are activated among others by inflammatory mediators, growth factors (PDGF and TGF-β1), cytokines (IL-1, IL-6, and IL-8), hormones, angiotensin II, intracellular signaling molecules, and reactive oxygen species (ROS) as well as hypoxia (Nielsen et al., 2017) and mechanical stimuli (Omary et al., 2007b; Fels et al., 2016, Fels et al., 2018; Ferdek and Jakubowska, 2017; Lachowski et al., 2017). Activated PSCs, in turn, secrete growth factors themselves so that they are engaged in a mutual positive feedback loop of other cells of the PDAC tissue (Fu et al., 2018). In addition, activated PSCs proliferate, migrate (Omary et al., 2007b), and secrete copious amounts of ECM components, especially collagen I and III (Ferdek and Jakubowska, 2017). The resulting changes in the pH values and increased stiffness of the desmoplastic tissue also feed back onto the behavior of PSCs (Lachowski et al., 2017). One of the mechanosensitive ion channels, Piezo1, that senses the mechanical properties of the PDAC microenvironment is inhibited by an acidic pH. This could prevent PSCs to be overridden by the mechanically triggered Ca^2+^ influx via Piezo1 channels (Kuntze et al., 2020).

### Ion Channels and Fibrosis

The function of ion channels in tumor stroma cells is far from being fully understood, especially regarding PDAC. Nonetheless, we already know that some ion channels play a significant role in the development of fibrosis in other organs such as K_Ca_3.1 in lungs, kidneys (Roach et al., 2013), and heart (Zhao et al., 2015); K_2P_2.1 in cardiac fibrosis (Abraham et al., 2018); and TRPV4 in liver (Songa et al., 2014), heart (Adapala et al., 2013), and lung fibrosis (Rahaman et al., 2014). Usually the inhibition of these ion channels attenuates the profibrotic response of the fibroblasts (Cruse et al., 2011; Adapala et al., 2013; Rahaman et al., 2014; Abraham et al., 2018; Roach and Bradding, 2019).

Ion channel research in PSCs is still in its infancy. We will therefore draw some analogies from hepatic stellate cells that are closely related to PSCs and in which these ion channels may play a similar role. Hepatic stellate cells are responsible for matrix homeostasis in healthy livers (Puche et al., 2013). Similar to the PSCs in PDAC, they are mainly responsible for the excessive production and remodeling of the ECM in the fibrotic liver (Puche et al., 2013; Freise et al., 2015; Ezhilarasan et al., 2018). For this reason, these types of cells have been suggested as a possible target for antifibrotic therapy.

**K**_**Ca**_ **3.1:** We do not have much information on the role of K_Ca_3.1 channels in PDAC-associated fibrosis, which is largely driven by PSCs. It is only known that K_Ca_3.1 channels regulate migration of PSCs (Storck et al., 2017).

So far, it is under debate whether K_Ca_3.1 has pro- or antifibrotic effects in the liver (Roach and Bradding, 2019). K_Ca_3.1 expression is increased in hepatic stellate cells after the incubation with TGF-β, a known activator of hepatic stellate cells. In both *in vitro* and *in vivo* experiments, the inhibition of K_Ca_3.1 shows an antifibrotic effect and decreases the expression of profibrotic genes (Freise et al., 2015). On the contrary, in the work of Møller et al., the inhibition or the absence of K_Ca_3.1 in hepatic stellate cells and hepatocytes worsens liver fibrosis (Møller et al., 2016). This information highlights the possible problems that ion channel therapies could face; the inhibition of an ion channel expressed in different cell types could have different effects.

**K**_**2P**_ **2.1:** So far, we only know that PSCs express K_2P_2.1 (previously designated as TWIK-related potassium channel-1; TREK1) (Fels et al., 2016). In fact, K_2P_2.1 is a mechanosensitive ion channel that can be modulated by pressure and membrane stretch (Lauritzen et al., 2005; Honoré, 2007) but also by pH. K_2P_2.1 contributes to setting the resting membrane potential of the cells (Bittner et al., 2014), and it is strongly correlated with proliferation and cell cycle in some tumors (Pethő et al., 2019). The mechanosensitive function of K_2P_2.1 is postulated to be involved in the migration, especially in the coordination of the front and rear ends of the cells (Pethő et al., 2019). Sauter et al. observed that the activation of K_2P_2.1 with BL 1249 in a PDAC line, BxPC-3, inhibits cell proliferation and migration through the hyperpolarization of the membrane (Sauter et al., 2016). Controversially, the absence of K_2P_2.1 in heart myofibroblasts from pressure-overloaded mice attenuates cardiac fibrosis also by decreasing fibroblast proliferation and migration (Abraham et al., 2018). This highlights again how the same ion channel can have a different impact on the behavior of different cell types and how this topic must be considered during the development of new therapies. However, it may also be seen as an indication that the “natural,” possibly fluctuating, activity is what matters physiologically. Clamping channel activity to a maximum or a minimum impairs cell function. It remains to be seen whether K_2P_2.1 channels exert a similar role in PDAC desmoplasia, where the unique tumor microenvironment could influence K_2P_2.1 function in many ways.

**TRPV4:** The transient receptor potential vanilloid channel 4 (TRPV4) is a mechanosensitive Ca^2+^-permeable nonselective cation channel that is expressed in many organs including the pancreas (Zhan and Li, 2018). TRPV4 is also expressed in PSCs. Its mRNA expression strongly decreases in PSCs when they are cultured under an elevated ambient pressure (+100 mmHg), mimicking the conditions that can be found in PDAC (Fels et al., 2016; Pethő et al., 2019; Sharma et al., 2019). The functional implications of this mechanosensitive expression have not yet been published. The decreased TRPV4 mRNA expression upon mechanical stimulation can be explained as a compensatory response of the cells which prevents Ca^2+^ overload following the pressure stimulus (Fels et al., 2016).

Notably, PSCs also release TGF-β upon stimulation with pressure (Sakata et al., 2004; Fels et al., 2016). TRPV4 integrates mechanical stimuli and soluble signals such as TGF-β, and it drives the epithelial–mesenchymal transition (EMT) (Adapala et al., 2013; Sharma et al., 2019). TRPV4 expression is dramatically increased in many tissue samples of patients with liver fibrosis (Songa et al., 2014). Furthermore, TRPV4 is highly expressed in hepatic stellate cells (Songa et al., 2014). Inhibition of TRPV4 decreases cell proliferation of hepatic stellate cells, decreases their TGF-β–dependent activation and the expression of collagen α1 and α-smooth muscle actin genes in *in vitro* cultures (Songa et al., 2014). Inhibition of TRPV4 also leads to an increase in apoptosis and inhibition of autophagy in the TGF-β–treated hepatic stellate cell line HCS-T6. These findings can be taken as indication for a similar role of TRPV4 channels in PSCs as well.

## Immunity and PDAC

The description/staging of cancers has significantly evolved over the last decades to include the tumor microenvironment (TME) and the infiltration of the tumors by the immune system (e.g., Immunoscore^®^ for colorectal cancers (Galon et al., 2014)). This is particularly important since T-cell infiltration, in general, bears a good prognostic feature: high CD4^+^ and CD8^+^ densities are associated with better overall and disease-free survival (Tang et al., 2014; Knudsen et al., 2017; Lohneis et al., 2017; Nejati et al., 2017).

The development of PDAC can be seen as a result of failed removal of malignant cells (Dunn et al., 2002). This failure might originate from the quantitative and qualitative composition of the immune cell repertoire in the TME, and/or altered function of the immune cells and their ion channels. An in-depth analysis of the immune cells in PDAC is beyond the scope of this review, and thus, we will use a simplified classification scheme and focus on the roles of the cells of innate and adaptive immunity in PDAC progression and how their roles may be modulated by ion channels. The expression of ion channels in immune cells in PDAC is summarized in Table 2. Addition of the immune component to a topical review on ion channels in PDAC is unique to this article, and thus, basic functions of immune cells have to be discussed briefly in the corresponding sections about a cell type.

**TABLE 2:** **Ion channel expression and function in innate and adaptive immune cells of pancreatic ductal adenocarcinoma.**

Channel	Function	Reference
**Neutrophils**		
K_Ca_3.1	Chemotaxis	Henríquez et al. (2016)
K_ir_2.1	Possible role in neutrophil proliferation, membrane potential regulation, and Ca^2+^ influx	Masia et al. (2015)
K_V_1.3	Membrane potential regulation and electric field detection	Kindzelskii and Petty (2005)
TRPC1	fMLF-stimulated migration and chemotaxis	Lindemann et al. (2015)
TRPC6	Chemotaxis and CXCL1-induced recruitment from the vasculature	Lindemann et al. (2013) and Lindemann et al. (2020)
TRPM2	*In vitro* transmigration	Yamamoto et al. (2008)
P2X7	IL-1β secretion	Karmakar et al. (2016)
H_V_1	Ca^2+^ entry regulation, ROS production, and neutrophil migration	El Chemaly et al. (2010)
		Ramsey et al. (2009)
**Monocytes/macrophages**		
K_Ca_3.1	M1 polarization	Xu et al. (2017)
K_2P_6.1	Inflammasome formation	Di et al. (2018)
TRPC1	M1 polarization	Chauhan et al. (2018)
TRPM2	Chemokine production	Yamamoto et al. (2008)
TRPM7	Ca^2+^-induced macrophage stimulation, proliferation, and M2 polarization	Schilling et al. (2014) and Schappe et al. (2018)
H_V_1	Phagosomal pH regulation and ROS production	El Chemaly et al. (2014)
**Dendritic cells**		
K_V_1.3, K_V_1.5	MHCII expression, migration, and cytokine production	Matzner et al. (2008)
Na_V_1.7	Migration	Zsiros et al. (2009)
P2X7	Antigen presentation and migration	Mutini et al. (1999) and Saéz et al. (2017)
H_V_1	ROS production	Szteyn et al. (2012)
**Myeloid-derived suppressor cells (MDSCs)**		
TRPV1	Promotes MDSC formation	Hegde et al. (2011)
P2X7	ARG-1, TGF- β1, and ROS up-regulation	Bianchi et al. (2014)
**NK cells**		
K_Ca_3.1	Negatively influencing proliferation, degranulation, and cytotoxicity	Koshy et al. (2013)
K_V_1.3	Positively influencing proliferation and degranulation	Koshy et al. (2013)
**CD4^+^ and CD8^+^ T‐cells**		
K_Ca_3.1	Sustaining Ca^2+^ influx during T-cell activation	Ghanshani et al. (2000) and Wulff et al. (2003)
K_V_1.3	Sustaining Ca^2+^ influx during T-cell activation	Wulff et al. (2003)
TRPM4	Motility and cytokine production	Weber et al. (2010)
CRAC^a^	Ca^2+^ influx during T-cell activation	Feske et al. (2012)
**T_reg_s**		
K_Ca_3.1	Still unclear	Estes et al. (2008)
K_V_1.3	Still unclear	Varga et al. (2009)
CRAC^b^	Development and differentiation	Vaeth et al. (2019)
**B cells**		
K_Ca_3.1	Sustaining Ca^2+^ influx during B-cell activation	Wulff et al. (2004)
K_V_1.3	Sustaining Ca^2+^ influx during B-cell activation	Wulff et al. (2004)
CRAC^c^	Ca^2+^ influx during B-cell activation	Feske et al. (2012)

^a^ Murine T‐cells: mRNA and fluorescence-based data indicate that T‐cells up-regulate *Orai1* and down-regulate *Orai2* when they become activated (Vaeth et al., 2017). The role of *Orai3* is controversial (McCarl et al., 2010; Vaeth et al., 2017).

Human peripheral T‐cells: the dominant isoform is *Orai1*, but all the three genes are up-regulated upon activation (Lioudyno et al., 2008). There is no difference in cell surface expression of ORAI1 between human memory and naive T‐cells (Cox et al., 2013).

^b^ Murine peripheral Tregs: mRNA data suggest the expression of *Orai1* and *Orai2*, while much less of *Orai3* (Vaeth et al., 2017).

Human peripheral T_reg_s: ORAI1 and ORAI2, but not ORAI3, were detected using immunocytofluorescence. The expression of *Orai1* in T_reg_s is significantly inferior compared to naive and activated CD4^+^ T‐cells (Jin et al., 2013).

^c^ Murine B cells express *Orai1*, *Orai2* and *Orai3* to a comparable extent (Gwack et al., 2008; Vaeth et al., 2017).

Human B cells: no detailed mRNA data. There is no difference in cell surface expression of ORAI1 between memory and naive B cells (Cox et al., 2013).

Based on the relative proportion of CD3^+^ and CD8^+^ cells over all cells in the tumor (Galon and Bruni, 2019), PDAC is often ranked among the “coldest” human tumors (Maleki Vareki, 2018). Although leukocytes (CD45^+^ cells) comprise almost 50% of all cells isolated from murine (Clark et al., 2007) and human PDAC (Trovato et al., 2019), T lymphocytes are significantly less abundant (15% of total cells in mice and ca. 20% in humans) compared to well-known “hot” tumors like melanoma (Sakellariou-Thompson et al., 2017; Blando et al., 2019). Low T-cell infiltration of PDAC can be due to a desmoplastic mechanical impediment, hypoxia, and low extracellular pH (Knudsen et al., 2017).

At the time of diagnosis, the TME is already highly immunosuppressive, which can be related to the high number of myeloid-derived suppressor cells in PDAC (Trovato et al., 2019). Moreover, the low pH and the alterations of the ionic composition of the TME may lead to the formation of tumor-associated immune cells which become the malfunctioning side of the immune response (Vesely et al., 2011; Gabrilovich et al., 2012; Girault et al., 2020). The fact that ion channels are expressed in both antitumor and protumor/suppressor immune cells allows us to consider ion channels as putative mediators of the biased immune response in PDAC (Feske et al., 2015; Fels et al., 2018).

### Ionic Composition of the Tumor Microenvironment

Distinct characteristics of PDAC, that is, poor vascularization and a markedly fibrotic stroma, result in deficient oxygen supply and metabolite accumulation (Olive et al., 2009; Provenzano et al., 2012). The high metabolic rate, glycolysis (GAPDH activity; production of lactate (Dovmark et al., 2017)), implementation of the pentose phosphate pathway, and production of CO_2_ are the source of protons which lead to extracellular acidification in PDAC (Gillies et al., 2002; Hashim et al., 2011; High et al., 2019). Such an acidification of poorly perfused tumor areas has a profound impact on the function of ion channels in all cells of the tumor tissue (reviewed in Pethő et al. (2020)). Acidification, severe hypoxia, and mechanical stress also cause cell necrosis. This is associated with an elevation of the [K^+^] in the interstitium (Cruz-Monserrate et al., 2014; Eil et al., 2016; Leslie et al., 2019). Moreover, the concentration of Na^+^, a major contributor of osmotic pressure in the interstitium, is increased, which can have multiple implications for the infiltration of immune cells (He et al., 2020). Thus, the ionic composition of the tumor microenvironment is characterized by altered concentration gradients across the plasma membrane, that is, by altered electrochemical driving forces and by constituents, for example, protons, which have a strong impact on channel activities. Importantly, the disrupted ionic composition is sensed by ion channels in cancer, immune, and stromal cells and inevitably affects their function. The consequences of the altered tumor environment on cell function through the modification of ion channels of immune cells will be discussed in the following.

### Cells of the Innate Immune Response

**Neutrophils:** A high number of neutrophils in the PDAC stroma is usually associated with poor prognosis (Wang et al., 2018; Oberg et al., 2019). Likewise, a high neutrophil-to-lymphocyte ratio (NLR), also in peripheral blood, is associated with a lower 5-year survival rate after tumor resection (Nywening et al., 2018).

Expression of voltage-gated and Ca^2+^-activated channels, K_V_1.3 and K_Ca_3.1, was shown in murine and human neutrophils (Krause and Welsh, 1990; Kindzelskii and Petty, 2005; Henríquez et al., 2016). Moreover, murine neutrophils express electrophysiologically detected inwardly rectifying K_ir_2.1 channels which are also assumed to contribute to their resting membrane potential and Ca^2+^ influx (Masia et al., 2015).

Since K^+^ channels are involved in neutrophil migration and chemotaxis like other cells present in the PDAC microenvironment, a high extracellular K^+^ concentration may also perturb neutrophil function. This assumption is important not only in the context of their ability to reach the cancer niche but may also be a cause of unfavorable retention of neutrophils in PDAC milieu.

While *intracellular* ATP is a regulator of neutrophils’ Kir6.x channels (Silva-Santos et al., 2002), *extracellular* ATP, for example, released from necrotic cells, induces neutrophil recruitment through purinergic P2X7 receptor activation (McDonald et al., 2010). Opening of the ATP-gated P2X channels leads to Ca^2+^/Na^+^ influx (Karmakar et al., 2016). Importantly, P2X7 is expressed also in cancer and PSCs, and the P2X7 inhibitor, AZ10606120, reduces cancer cell proliferation *in vitro* and *in vivo* (Haanes et al., 2012; Giannuzzo et al., 2015).

Ca^2+^ signaling plays a major role in neutrophil migration, phagocytosis, and ROS production. One of the key mechanisms in the Ca^2+^ increase is mediated by store-operated Ca^2+^ entry (SOCE) and subsequent activation of Orai1 channels. Several other Ca^2+^-permeable TRP channels are also involved in the innate immune response (Najder et al., 2018). Since neutrophils express C-X-C chemokine receptor–type 2 (CXCR2), they are attracted by ligands like CXCL1/IL-8, CXCL2, and CXCL5, released in pancreatitis and pancreatic cancer (Saurer et al., 2000; Steele et al., 2016; Najder et al., 2018; Wu et al., 2019; Zhang et al., 2020). Indeed, inhibition of CXCR2 signaling in PDAC shows beneficial results (Ijichi et al., 2011; Steele et al., 2016). Recruitment of neutrophils upon CXCR2 activation is mediated by Na^+^ and Ca^2+^-permeable, classical/canonical transient receptor potential 6 (TRPC6) channel (Lindemann et al., 2013, 2020). TRPC6 is also expressed in PSCs, where it mediates hypoxia-induced migration and production of cytokines (Nielsen et al., 2017). In a mouse model, it could be shown that inhibition of TRPC6 with specific antagonists (SAR7334, BI-749327) diminishes the inflammatory response in the lungs and ameliorates cardiac and renal fibrosis (Lin et al., 2019; Chen et al., 2020). One can presume that such a beneficial effect could also be elicited in PDAC, in part by inhibiting neutrophil recruitment into the tumor.

In colorectal cancer, another chemokine receptor, formyl peptide receptor (FPR1), is highly expressed in tumor-infiltrating, myeloperoxidase-positive (MPO^+^) cells (Li et al., 2017). Also, FPR1 is enriched in immune cells of the recently suggested L4 PDAC subtype (Zhao et al., 2018). In murine neutrophils, FPR1-mediated directed migration depends on TRPC1 channels which may therefore contribute to neutrophil infiltration in PDAC (Lindemann et al., 2015; Fels et al., 2018).

Once at the target, activated neutrophils produce ROS, release metalloproteinases (e.g., MMP-9) and cytokines, and form neutrophil extracellular traps (NETs) (Wu et al., 2019). The remarkable ability of neutrophils to produce ROS depends on the depolarizing activity of the NADPH oxidase (NOX2) and concomitant action of voltage-gated proton channels (H_V_1) (DeCoursey et al., 2016). Their activity in neutrophils is very relevant for PDAC progression. Neutrophil-derived ROS may cause cancer apoptosis due to TRPM2 channel activation (Gershkovitz et al., 2018). Accordingly, pharmacological stimulation of ROS production induces pancreatic tumor cell apoptosis (Shi et al., 2008). However, channel expression in cancer cells can also promote cancer cell proliferation (Lin et al., 2018). Release of NETs, a defense mechanism of extruding DNA covered with enzymes and histones, is often ROS-dependent and is therefore indirectly mediated by H_V_1 activity. NET formation can occlude pancreatic ducts, cause pancreatitis, and promote PDAC metastasis to the liver (Leppkes et al., 2016; Takesue et al., 2020). Also, distant PDAC metastasis is facilitated by activated neutrophils in the circulation (Tao et al., 2016). Thus, aiming at the H_V_1 channel in cancer therapy could have potential benefits, mostly due to inhibition of ROS-related activity of neutrophils (Fernández et al., 2016).

**Macrophages:** Tumor-associated macrophages (TAMs) are generally divided into “classically activated” M1 and “alternatively activated” immunosuppressive M2 macrophages. The latter type is predominant in PDAC tissue (Habtezion et al., 2016; Hu et al., 2016; Liu et al., 2016). M2 polarization is induced by IL‐4 and IL‐13 (Biswas and Mantovani, 2010). The presence of these cells in PDAC is associated with poor prognosis. TAMs also contribute to formation of desmoplasia through interplay with PSCs and mutual stimulation of cytokine production. Moreover, macrophage-derived metalloproteinases mediate dynamic turnover of fibrotic tissue and allow for tumor expansion (reviewed in Hu et al. (2015)). Chemotherapy can induce macrophage polarization into the tumoricidal M1 type and improve therapy outcome (Kurahara et al., 2011; Di Caro et al., 2015). Macrophage recruitment to the tumor site is mediated by CCL2/CCR2 and CSF-1/CSF-1R axes, with the latter additionally promoting M2 polarization. Inhibition of these signaling pathways shows potential benefits so that the CCL2/CCR2 inhibitor (PF-04136309) is implemented in PDAC clinical trials (NCT01413022) (Zhu et al., 2014; Habtezion et al., 2016; Nywening et al., 2018).

Channels expressed in macrophages often overlap with those expressed in neutrophils, especially in regard to K^+^ channels (reviewed in: Feske et al. (2015)). There is also evidence that K_2P_6.1 (TWIK2) mediates K^+^ efflux. In murine macrophages, K_2P_6.1 leads to inflammasome formation and—in cooperation with the depolarizing action of P2X7—induces release of IL-1β (Di et al., 2018).

Ca^2+^ signaling plays a crucial role for macrophage function. Thus, migration and phagocytosis of macrophages depend on Ca^2+^-permeable channels (Desai and Leitinger, 2014). In addition, the polarization of macrophages is mediated by several Ca^2+^-permeable ion channels including few members of the TRP channel family. TRPM7 promotes M2 polarization and shows high activity in this type of macrophage (Schilling et al., 2014). In contrast, deletion of TRPM2 favors a proinflammatory macrophage phenotype in *Helicobacter pylori* infection (Beceiro et al., 2017). M1 macrophage polarization is promoted by the activity of TRPC1 and K_Ca_3.1 (Xu et al., 2017; Chauhan et al., 2018). These channels could be taken under consideration in approaching macrophage plasticity in PDAC, since M2 macrophages comprise the majority of infiltrated immune cells. However, since TRPC1 and K_Ca_3.1 channels are expressed not only in neutrophils but also in cancer and stromal cells as well as in lymphocytes, the impact of activating these channels is difficult to predict (see below, *Pharmacological Targeting of Ion Channels in PDAC* for a more detailed discussion).

**Dendritic cells:** There are a few dendritic cells at the tumor site and in the circulation of PDAC patients. The ability of dendritic cells to present foreign antigens has been used for designing dendritic cell–based immunotherapy (dendritic cell vaccines) against pancreatic cancer (Deicher et al., 2018). Some data indicate that dendritic cells predominantly support immunological tolerance in the strongly immunosuppressive PDAC environment (Barilla et al., 2019). Encountering an antigen elicits [Ca^2+^]_i_ to rise in dendritic cells, which is mediated by CRAC channels. The voltage-gated K^+^ channels, K_V_1.3 and K_V_1.5, modulate Ca^2+^ fluxes by hyperpolarizing the membrane potential. They are involved in major histocompatibility complex II expression, migration, cytokine production, and phagocytosis (Matzner et al., 2008). P2X7 is also expressed in murine dendritic cells, mediating antigen presentation and migration (Mutini et al., 1999; Saéz et al., 2017). However, despite their crucial role in coordinating the immune response, the involvement of ion channels in functions of dendritic cells present in PDAC tissue is not yet well-described.

**Myeloid-derived suppressor cells:** Myeloid-derived suppressor cells (MDSCs) are not fully differentiated myeloid cells which exhibit highly immunosuppressive features. They can be further divided into polymorphonuclear (PMN-MDSC) and monocytic (M-MDSC) in mice, and early-stage (eMDSC) MDSCs in human. They share some phenotypic features with differentiated myeloid cells but can be distinguished by their inhibitory properties (Trovato et al., 2019). PSCs are presumed culprits of promoting MDSCs in pancreatic cancer *via* IL‐6 release (Mace et al., 2013). MDSCs themselves exhibit increased arginase 1 activity, depleting the tumor microenvironment of L-arginine, which, in turn, elicits T-cell suppression. Not surprisingly, MDSC depletion is a looked for method for PDAC treatment (Thyagarajan et al., 2019).

There is an immense lack of knowledge about the function of ion channels in MDSCs. P2X7 and TRPV1 are the only channels described in MDSCs so far. P2X7 activation in M-MDSCs increases arginase-1, TGF-β, and production of ROS (Bianchi et al., 2014). In mice, TRPV1 activation stimulates MDSCs and protects from hepatitis (Hegde et al., 2011). The role of MDSC ion channels in the PDAC environment still needs to be elucidated.

Natural killer cells: Natural killer cells (NK cells) are innate lymphoid cells, and their function is similar to that of cytotoxic CD8^+^ cells. Despite the fact that their percentage ranges around 1.5–2% of mononuclear cells (Marsh et al., 2014; Bazhin et al., 2016) (which becomes 5% of leukocytes after a partial resection (Gürlevik et al., 2016)), their role is important. The intravenous injection of an NK cell line (LNK) into the tumor improves the survival of mice and delays PDAC growth (Hu et al., 2019b). Several clinical trials built on NK cell–based immunotherapy are on at the moment (Sunami and Kleeff, 2019).

The role of NK cells against cancer is now well documented, and their ion channels appear to be of pivotal importance (Redmond and Buchanan, 2017). Like essentially all other lymphoid cells, NK cells have CRAC currents and K^+^ currents mediated by K_V_1.3 and K_Ca_3.1, which are crucial for their function (Redmond and Buchanan, 2017). Koshy et al. discovered that a minority of human NK cells, defined as adherent NK cells, is able to nearly double the number of their K_V_1.3 and K_Ca_3.1 channels after their activation by cocultured cancer cells (K_V_1.3: 50 to 125/cell; K_Ca_3.1: 20 to 40/cell). Contrariwise, the majority of NK cells, named “nonadherent,” up-regulate only K_V_1.3, while K_Ca_3.1 channels remain unaltered (K_V_1.3: 20 to 350/cell; K_Ca_3.1: 20 to 15/cell) (Koshy et al., 2013).

### Cells of the Adaptive Immune Response

**FIGURE 2:**
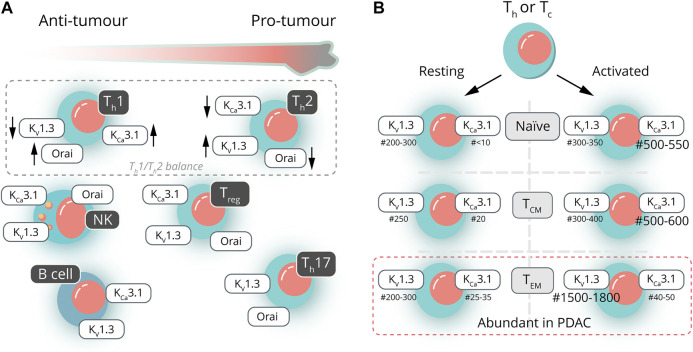
Ion channels in pancreatic ductal adenocarcinoma—infiltrating lymphocytes. **(A)** Lymphocytes found in the PDAC microenvironment can be either pro- or antitumorigenic. The ratio of T-helper lymphocytes (T_h_1/T_h_2; dashed line) has a prognostic value in assessing therapy outcome. The presence of other lymphocytes can be either beneficial (NK and B cells) or detrimental (T_reg_ and T_h_17) (yet, not univocally). These PDAC-infiltrating lymphocytes often show distinct channel activities, which could be considered in targeted PDAC therapies. **(B)** Both helper (T_h_) and cytotoxic (T_c_) T lymphocytes can be further subdivided into naive, central memory (T_C,M_) or effector memory (T_EM_) T lymphocytes, the latter being the most abundant subtype in PDAC (indicated by the dashed line). Activation of T lymphocytes leads to characteristic changes in the numbers (#) of K_Ca_3.1 and K_V_1.3 channels. Activation of naive and T_CM_ is associated with an increase in K_Ca_3.1 expression, whereas activation of T_EM_ causes a distinct increase in the number of K_V_1.3 channels.

The PDAC tissue is heavily infiltrated by different subsets of T‐cells and B cells. Depending on the nature of the cells and the cytokines being secreted, these cells can be both protumoral and antitumoral. Unfortunately, very little information is available about the ion channel expression of the different T-cell subsets in PDAC. To set the frame for future research on ion channels in PDAC-associated T- and B-cell subsets, we will first summarize the general scheme about the dependence of T-cell activation on ion channels and then focus on the T- and B-cell subsets relevant in PDAC (Figure 2A) along with mostly non-PDAC–specific information available about the ion channel expression of those T- and B-cell subsets. Figure 2B provides an overview with respect to the expression (changes) in two of the most important K^+^ channels found in the various subtypes.

#### Adaptive Immunity: Antitumor Cells

**Principal ion channels in T‐cells.** The function of ion channels in T lymphocytes has been thoroughly investigated in the last three decades (Cahalan and Chandy, 2009). T lymphocyte activation strictly depends on extracellular Ca^2+^ entry via Ca^2+^ release–activated channels, CRAC, composed of Orai (three homologues: *Orai1*, *Orai2*, and *Orai3*) and Stim (two homologues: *Stim1* and *Stim2*) proteins (Feske et al., 2012). In human, T lymphocyte Orai1 is essential for correct T-cell functioning (Vaeth et al., 2017). Ca^2+^ entry is facilitated by the opening of two K^+^ channels, the voltage-gated gated K_V_1.3 and the Ca^2+^-activated K_Ca_3.1, which is activated by the increase in the cytosolic free Ca^2+^ concentration above 200 nM (Panyi et al., 2014). Like in PDAC cancer cells (see *K* ^*+*^ *Channels in Pancreatic Cancer Cells*), these K^+^ channels maintain a permissive negative membrane potential for efficient Ca^2+^ signaling, and their inhibition interferes with T-cell activation (Panyi et al., 2006). This general mechanism is tailored to the T-cell subtypes either by changing the relative expression level of K_V_1.3 vs. K_Ca_3.1, or by changing the subunit composition of the CRAC channel.

**T-cell subsets in PDAC and their corresponding ion channel repertoire.** The major antitumor *effector* cells in PDAC belong to CD4^+^ helper and CD8^+^ cytotoxic cells, usually present in the tumor mass in similar proportions (Carstens et al., 2017; Stromnes et al., 2017). CD4^+^ helper T‐cells are actually a broad composition of several subtypes (mainly T_h_1, T_h_2, and T_h_17). The 20-year-old theory of the so-called “T_h_1/T_h_2 balance” stated that the cancerous environment causes a decrease in the T_h_1/T_h_2 ratio, toward a T_h_2-dominated and protumoral condition (Shurin et al., 1999). Also in PDAC, the helper T-cell ratio is pivotal (Wörmann et al., 2014). Thus, T_h_2-related cytokines like IL-4 (Piro et al., 2017) or IL-6 (Mroczko et al., 2010) have been deemed as “prognostic” by many researchers (De Monte et al., 2011; Yako et al., 2016). T_h_17 cells have an uncertain position in the tumor milieu, although the majority of cases tends toward a protumoral effect (Murugaiyan and Saha, 2009; Ivanova and Orekhov, 2015). IL-17 and IL-22, produced by T_h_17 cells, are correlated with a bad prognosis in PDAC (McAllister et al., 2014; Wen et al., 2014). Contrariwise, using the murine cancer cell line PANC-02 and inducing T_h_17 function produced an antitumor effect (Gnerlich et al., 2010).

When naive T‐cells encounter their specific antigens, they differentiate into central memory T‐cells (T_CM_) and effector memory T‐cells (T_EM_) of either CD4^+^ or CD8^+^ phenotype. The majority of T‐cells in an orthotopic mouse PANC02 PDAC model are effector/effector memory T‐cells (Shevchenko et al., 2013; Bazhin et al., 2016). In a similar fashion, in human PDAC, most CD8^+^ tumor-infiltrating lymphocytes are effector memory cells (Poschke et al., 2016; Stromnes et al., 2017).

As noted above, relatively little is known about the ion channel expression pattern in various T-cell subsets infiltrating the PDAC tissue. Compared to naive T‐cells, rich in both Orai1 and Orai2, effector CD4^+^ and CD8^+^ T‐cells down-regulate only Orai2, generating more Orai1 homohexamers, which are characterized by a superior Ca^2+^ conductance. In this manner, effector T‐cells allow larger Ca^2+^ influxes and activate quicker after the antigen recognition than naive cells (Vaeth et al., 2017). Naive human CD4^+^ and CD8^+^ T lymphocytes are characterized by 200–300 K_V_1.3 and less than 10 functional K_Ca_3.1 channels, whereas their activated counterparts mildly up-regulate K_V_1.3 (300–350 channels/cell) and severely increase K_Ca_3.1 expression (500–550 channels/cell) (Ghanshani et al., 2000; Wulff et al., 2003; Cahalan and Chandy, 2009).

Murine CD4^+^ T_h_1 and T_h_2 have similar numbers of K_V_1.3 channels in their membranes, while murine T_h_2 cells have substantially less functional K_Ca_3.1 channels than T_h_1 lymphocytes. This difference in the K_Ca_3.1 expression/function may explain the larger amplitudes of the Ca^2+^ signals in T_h_1 cells (Fanger et al., 2000; Di et al., 2010). A flow cytometric analysis of K_V_1.3 expression in human peripheral blood lymphocytes showed that the T_h_1 subset has less K_V_1.3 channels than T_h_2 (Toldi et al., 2011; Orbán et al., 2014). Consistent with this, they also reported that K_V_1.3 inhibitors have a smaller impact on the Ca^2+^ transients in T_h_1 lymphocytes than K_Ca_3.1 inhibitors (Toldi et al., 2011). Moreover, Ca^2+^ influx through CRAC is more prevalent in T_h_1 than T_h_2 cells (Toldi et al., 2012). Both mouse and human T_h_17 lymphocytes have the highest K_V_1.3 expression, but no or little K_Ca_3.1 expression (Di et al., 2010; Orbán et al., 2014). It should be pointed out that the role of K_V_1.3 and K_Ca_3.1 in regulating Ca^2+^ signaling in human T-cell subsets, addressed in the articles above, has not yet been confirmed using electrophysiology.

T_CM_ and T_EM_ memory cells not only differ in characteristic membrane markers and in their homing and trafficking ability but also in their ion channel repertoire. Human resting T_CM_ and T_EM_ cells, whether they are CD4^+^ or CD8^+^, are similar to naive cells regarding their K_V_1.3 and K_Ca_3.1 expression. When activated, they dichotomically diverge: T_CM_ cells show the usual K_Ca_3.1^high^ phenotype, whereas T_EM_ cells exhibit a dramatically increased K_V_1.3 expression in the plasma membrane (Ghanshani et al., 2000; Wulff et al., 2003; Cahalan and Chandy, 2009). (For more details with respect to the channel expression of the various subtypes, see Figure 2B.)

**B lymphocytes in PDAC and their corresponding ion channel repertoire.** The role of B cells in the pathology of PDAC is not well defined. In human PDAC, they are associated with a good prognosis (Castino et al., 2016; Brunner et al., 2020). However, this is not adequately mirrored by the existing mouse models (Spear et al., 2019). In 2016, three different research groups showed that B cells have a protumorigenic role in genetically modified mice (KC mice expressing the oncogenic Kras in the pancreas only) and in healthy mice orthotopically injected with KC cells (reviewed in Roghanian et al. (2016)). KMC, a mouse model characterized by the knock-in of one or two copies of *Myc* (Farrell et al., 2017), develops one of the most aggressive and histologically human-like PDAC. It is not as strongly infiltrated by NK and B cells as the slowly developing KC model. The removal of the *Myc* gene promotes NK and B cells to enter the tumor mass, lengthening the life span of mice (Muthalagu et al., 2020). Some studies merely report B-cell infiltration in PDAC based on their CD20 expression (Brunner et al., 2020), and further classification of the cells based on their activation status (i.e., CD27 expression) is lacking. Other studies, for example, Castino et al., found that interspersed B cells from PDAC show little or no CD27 and other fundamental markers. However, when they organize themselves in tertiary lymphoid tissue structures within the tumor stroma, CD27 expression is up-regulated (Castino et al., 2016).

There is a strong relationship between the ion channel expression and the activation status of the B cells (Wulff et al., 2004). Human naive (IgD^+^ CD27^−^) and early memory (IgD^+^ CD27^+^) B cells, just like naive and central memory T‐cells, are abundant in K_V_1.3 and virtually lack K_Ca_3.1 (naive: K_V_1.3: 90–100, K_Ca_3.1: 5 channels/cell; early memory: K_V_1.3: 250, K_Ca_3.1: 5–10 channels/cell). These cells, when activated, overexpress only K_Ca_3.1 channels (naive: K_V_1.3: 80–100, K_Ca_3.1: 550–650 channels/cell; early memory: K_V_1.3: 150–200; K_Ca_3.1: 650–750 channels/cell) (Wulff et al., 2004; Cahalan and Chandy, 2009). Late memory class-switched B cells (IgD^−^CD27^+^) have plenty of K_V_1.3 and few K_Ca_3.1 (K_V_1.3: 2,200–2,600; K_Ca_3.1: 50–70 channels/cell). They tend to further enhance their K_V_1.3 expression when activated (K_V_1.3: 2,900–3,300; K_Ca_3.1: 60–80 channels/cell).

#### Adaptive Immunity: Protumor Cells

**Regulatory T‐cells in PDAC and their corresponding ion channel repertoire.** Human T_reg_ cells are important immunosuppressive CD4^+^ lymphocytes heavily implicated in autoimmunity. They are usually identified by the signature CD4^+^CD25^+^Foxp3^+^ (Whiteside, 2015). In contrast to the healthy pancreas (Weisberg et al., 2019), PDAC is heavily infiltrated by T_reg_s. They can reach 20–40% of the whole CD4^+^ pool (Shevchenko et al., 2013; Tang et al., 2014; Bazhin et al., 2016). Recently, it has been discovered that PDAC tissues overexpress a cancer-related Foxp3 protein (c-Foxp3) which, through the secretion of CCL5, would recruit a high number of T_reg_ cells in the tumor environment (Wang et al., 2017).

Similarly to their helper CD4^+^ counterpart, human T_reg_s also have a high number of voltage-gated K_V_1.3 channels (Estes et al., 2008; Shao et al., 2018) and a low number of K_Ca_3.1 channels in their membrane (Varga et al., 2009). Intratumoral T_reg_s have a peculiar T-cell receptor repertoire whose stimulation may have an important role in their immunosuppressive function (Ahmadzadeh et al., 2019). When human T_reg_s get activated by T-cell receptor stimulation, they do not up-regulate K_V_1.3 as normal effector T‐cells do (Reneer et al., 2011) and activated human T_reg_s incubated with K_V_1.3 and K_Ca_3.1 blockers do not show any difference in the Ca^2+^ influx, suggesting that the contribution of these channels to the activation could be minimal (Orbán et al., 2014). Moreover, T_reg_s from K_Ca_3.1 knockout mice are able to suppress T-cell proliferation in a comparable manner as wild‐type T_reg_s, pointing to a minor role of these channels (Di et al., 2010). Similarly, knocking out K_V_1.3 in mice does not hinder the physiological generation of T_reg_s (Gocke et al., 2012). Hence, the role of these K^+^ channels in T_reg_s is currently still unclear. CRAC channels are involved in T_reg_ development and contribute to their suppressive function (Vaeth et al., 2019).

## Therapeutical Approaches and Ion Channels in PDAC

Unfortunately, PDAC therapy has remained largely ineffective. Radical surgical resection of the tumor as well as chemotherapeutic agents like gemcitabine combined with nab-paclitaxel and FOLFIRINOX constitutes the standard therapy for PDAC patients (Hessmann et al., 2020). However, 80–90% of the patients present at an advanced unresectable stage at the time of diagnosis. Even if surgical intervention is possible, recurrence of the cancer lesions will be common (Vincent et al., 2011; Peixoto et al., 2015; Rawla et al., 2019). Therapy resistance is in part due to the fibrotic microenvironment in PDAC which hinders drugs from reaching their target and due to the immunosuppressive properties of the PDAC tumor microenvironment. Nonetheless, so far, therapeutic targeting of the PDAC tumor microenvironment has not been successful (reviewed in Hessmann et al. (2020)).

### Electrolytes and Organic Metabolites in PDAC

The great majority of the studies using ion channel blockers or activators are aimed to target cancer cells, rather than immune or stroma cells. The therapeutic potential of ion channels of cancer-associated immune and stroma cells has not been analyzed in great detail so far. In a groundbreaking study by Eil et al., it was shown that B16 (mouse) or Mel624 (human) melanoma cells subcutaneously injected into mice create a TME much richer in K^+^ than serum (40 mM vs. 5 mM), probably due to marked necrosis within the tumor (Eil et al., 2016). Since more than 60% of PDAC cases host micro- and/or macronecrotic spots (Hiraoka et al., 2010), it is plausible to hypothesize that PDAC is a tumor rich in extracellular K^+^ as well. The T‐cell [K^+^]_i_ is around 130 mM, but when cultured in a medium with high [K^+^]_o_—specular of what happens *in vivo*‐ the [K^+^]_i_ in these cells can rise above 150 mM (Eil et al., 2016; Ong et al., 2019). Although the [K^+^] changes would result in the depolarization of the cell membrane, the authors did not find a diminished Ca^2+^ influx into the T‐cells during their activation in high [K^+^]_o_ (Eil et al., 2016). This is opposite to the generally accepted role of the membrane potential in controlling Ca^2+^ signaling.

Membrane depolarization could also lead to enhanced IL-2 signaling in T_reg_s and, consequently, to suppressed antitumor immune surveillance by such an unbalanced ionic environment (Nagy et al., 2018). The high [K^+^]-adapted tumor-infiltrating lymphocytes are less functional than the normal ones. Conversely, decreasing the [K^+^]_i_ in a forced manner using the Na^+^/K^+^ ATPase blocker ouabain renders the CD8^+^ cells more functional again. Moreover, substituting normal (poorly functioning) immune cells with CD8^+^ cells overexpressing the K^+^ efflux channels K_V_1.3 or K_Ca_3.1 boosts their antitumor activity. Tumor growth is slowed down, and this improves the survival (Eil et al., 2016). These findings nicely illustrate how ion channel function and thereby cell function depend on the “correct” ionic composition of the pericellular environment.

The disrupted ion balance in the tumor microenvironment also affects the operation of protumor immune cells. Increasing [Na^+^]_o_ and [Cl^−^]_o_ in melanoma as well as lung and breast cancer by the administration of a high salt diet (HSD) inhibits the capacity of MDSCs to suppress antitumor cytotoxic cells. Accordingly, this treatment has made the tumors to shrink in size (Willebrand et al., 2019; He et al., 2020). High [Na^+^]_o_ and [Cl^−^]_o_ partially inhibit the function of thymus-derived mouse T_reg_s as well (Luo et al., 2019), which could also contribute to the less immunosuppressive environment. Whether these electrolytes influence antitumor CD4^+^ and CD8^+^ T‐cells is unclear. Melanoma and breast cancer growth in BABL/C-nu/nu mice, lacking sufficient T-cell–mediated immune reactions, are insensitive to a high salt diet, indicating that T-cell–mediated antitumor response is key to the high salt diet–induced antitumor activity (He et al., 2020). On the other hand, the high salt diet prevents tumors from growing also in RAG2^−/−^ mice, despite the lack of T and B cells in these animals. This suggests that the high salt diet may act by yet another mechanism independent of immune cell modulation (Willebrand et al., 2019). All these studies clearly show that the electrolyte imbalance in the tumor microenvironment significantly contributes to tumor progression and may shape the immune response.

High salt diet increases the osmolarity in the cancer tissue (He et al., 2020), which may influence the volume regulation of immune and cancer cells and link the high–salt diet effects described above to ion channels. Cell volume changes are known to regulate cancer cell migration, invasion, and apoptosis in an ion channel (and transporter)–dependent manner (see for reviews (Bortner and Cidlowski, 2014; Schwab and Stock, 2014)). In addition, cell volume changes in immune cells regulate apoptosis (Bortner and Cidlowski, 2011) and B-cell activation (Cvetkovic et al., 2019). Although the pivotal role of ion channels in volume regulation in immune (Feske et al., 2015) and cancer cells (Morishita et al., 2019) is well established, the relevant ion channels that sense the altered ion concentrations and osmolarity in the PDAC microenvironment are yet to be identified.

In addition to [K^+^], the pH in the tumor microenvironment also changes characteristically during the progression of the disease. The nondiseased pancreas stroma is deemed to endure postprandial tides of acidic pH in order to counterbalance the apical excretion of HCO_3_^−^ ions. During the development of PDAC, the tumor microenvironment becomes very acidic (Pedersen et al., 2017). The extra- and intracellular pH regulate voltage-gated K^+^ channels of the K_V_1 Shaker family (Starkus et al., 2003). One of the major K^+^ channels in lymphocytes, K_V_1.3, in particular, is uniquely modulated by acidification. A decrease in both pH_i_ and pH_o_ reduces the peak K_V_1.3 current, and acidification of the extracellular medium slows inactivation kinetics of the current (Deutsch and Lee, 1989). This dual regulation of K_V_1.3 by pH_o_ allows very sensitive modulation of the K^+^ conductance of the membrane. Acidification-induced K^+^ current inhibition is counterbalanced, depending on pH_o_, by the concomitant slowing of the inactivation kinetics. Interestingly, the slowing of the inactivation kinetics at low pH_o_ is reverted to acceleration of the kinetics when acidic extracellular pH is combined with elevated [K^+^]_e_ (Somodi et al., 2008). The unique response of K_V_1.3 to extracellular acidification is mediated by reversible protonation of H399 in the external vestibule of the channel guarding the selectivity filter (Somodi et al., 2004). The protonation of H399 also bears significance on the targeting of the K_V_1.3 channel by peptide and nonpeptide inhibitors as well. For example, tetraethylammonium (Somodi et al., 2004) and peptide blockers (Rodrigues et al., 2003) lose their affinity for K_V_1.3, when H399 is protonated. This molecular information about K_V_1.3 gating at various pH and K^+^ concentrations may contribute to the understanding of how the tumor microenvironment interacts with immune cells at the level of ion channels and may allow proper therapeutic ion channel targeting. We refer to our recent review for a more detailed discussion on how cancer progression may be affected by modulation of cation channels in tumor and tumor stroma cells (Pethő et al., 2020).

The disbalance of the tumor microenvironment is not restricted to inorganic electrolytes. It is well known that tumors are rich in ATP and adenosine, which influence cells and their function through purinergic receptors (Feng et al., 2020). The ATP concentration in the interstitial tissue of PDAC is almost 100 times higher than normal pancreatic stroma (∼10 μM vs. 100 nM (Hu et al., 2019a)). PDAC cells usually overexpress both CD39 (Künzli et al., 2007) and CD73 (Harvey et al., 2020), which transform ATP to AMP and AMP to adenosine. Thus, it is plausible that PDAC is rich in adenosine as well, which would activate the receptor A_2A_R and inhibit CD8^+^ cells via PKA (Chimote et al., 2013). As more thoroughly described later, adenosine also decreases the K_Ca_3.1 conductance of T‐cells without affecting the number of channels in the membrane (Chimote et al., 2018). Adenosine exerts *in vivo* and *in vitro* an anti-PDAC effect in immunodeficient nu/nu mice (Yang et al., 2019). This means that the outcome of the adenosine action will be the combination of inhibiting antitumor immunity and the growth of PDAC. Three clinical trials are currently assessing whether antiadenosine therapy coupled with immuno- and chemotherapies is able to halt PDAC progression (Singh and O’Reilly, 2020).

### Pharmacological Targeting of Ion Channels in PDAC

Ion channel targeting is a well-established therapeutic concept in other medical disciplines that has been successfully applied in the clinical routine for decades. Because of their expression in the plasma membrane, ion channels are easily druggable. Intriguingly, many of the ion channels that are known to drive tumor progression are targeted by drugs currently used for noncancer indications, either as the primary effect or as a side effect. These drugs could in principle be repurposed for cancer treatment as suggested for nontorsadogenic K_V_11.1 blockers (Kale et al., 2015; Pointer et al., 2017). The fact that cancer, stroma, and immune cells rely nearly on the same set of ion channels makes the selection of the right drug a complex quest. Ideally, channel targeting should elicit synergistic effects such as inhibition of tumor and stoma cell proliferation or migration as well as activation of antitumor immune cells or inhibition of tumor-promoting immune cells. This is further complicated by the dynamics/variability of channel expression: in lymphocytes, for example, such expression strongly relies on the activation state of the respective subtype. Moreover, specificity of the channel modulators constitutes another challenge which has not been solved satisfactorily for many channels. However, an increasing number of ion channel protein structures is becoming amenable for *in silico* drug design (e.g., Brömmel et al. (2020b)). Alternatively, peptide-based blockers or antibody targeting could be more specific alternatives (Duranti and Arcangeli, 2019; Hartung et al., 2020; Tajti et al., 2020). Here, we list potential target ion channels and the existing knowledge about the consequence of their activation/inhibition in immune, stroma, and tumor cells as well.

**K**_**Ca**_ **3.1** is a well-studied channel in this context. The specific modulators of K_Ca_3.1 are inhibitors, such as TRAM-34 (Wulff et al., 2000), senicapoc, NS6180, and verapamil (Wulff et al., 2007), and activators such as riluzole (Liu et al., 2013) and 1-EBIO (Devor et al., 1996). Molecular modeling suggests that senicapoc binds to K_Ca_3.1 channels in the open conformation (Brömmel et al., 2020a). The inhibition or activation of an ion channel may result in diminished or augmented cellular functions. For example, two specific inhibitors of K_Ca_3.1 (TRAM-34 and NS6180) boosted *in vivo* adherent human NK-cell proliferation, degranulation, and capacity of killing erythroleukemic cells in mice (Koshy et al., 2013). Conversely, activation of K_Ca_3.1 channels with 1-EBIO inhibits migration of transformed renal epithelial cells almost as effectively as channel inhibition (Schwab et al., 2006). As discussed above, T lymphocytes in PDAC are supposedly fairly devoid of K_Ca_3.1; hence, the use of such a blocker should not hinder physiological function of these cells, regardless of [K^+^] in the tumor microenvironment. On the other hand, in mice, the adoptive cell transfer of T_CM_ cells, a minority in PDAC but rich in K_Ca_3.1, more efficiently combats melanoma than the transfer of the T_EM_ subset that poorly expresses K_Ca_3.1 (Klebanoff et al., 2005, 2016). Unfortunately, the consequence of the modulation of K_Ca_3.1 activity of T_EM_ and T_CM_ cells has not been elaborated yet in tumor models.

K_Ca_3.1 is unique among the K^+^ channels of the immune system since the consequences of both channel inhibition and activation can be studied. Activation of K_Ca_3.1 by 1-EBIO is able to induce movement in akinetic CD8^+^ cells isolated from the peripheral blood of head and neck squamous cell carcinoma patients and ameliorate their IFN-γ production (Chimote et al., 2018). Thus, pharmacological activation of K_Ca_3.1 may enhance antitumor activity of CD8^+^ T‐cells by overcoming the inhibition of the K_Ca_3.1 activity caused by large amounts of adenosine in cancer stroma and/or localized down-regulation of membrane-proximal calmodulin, the Ca^2+^ sensor of K_Ca_3.1. The diminished association of calmodulin with the channel suppresses the K_Ca_3.1 activity in circulating T‐cells and limits their ability to infiltrate adenosine-rich tumor-like microenvironments (Chimote et al., 2020). How NK cells would behave with a K_Ca_3.1 activator has not been studied. It is known however that in patients suffering from amyotrophic lateral sclerosis, riluzole, which is the only disease-modifying therapy for ALS, does not compromise NK cells from entering the spinal cord and the motor cortex (Garofalo et al., 2020). Based on this, we can hypothesize that K_Ca_3.1 activation would not interfere with the antitumor action of NK cells.

*In vitro* studies also demonstrated that the K_Ca_3.1 activator SKA-346 increases the IFN-γ secretion of high [K^+^]-inhibited human T‐cells by 50% (Ong et al., 2019). Neither this nor new K_Ca_3.1 activators, like SKA-31 and SKA-121 (Ohya and Kito, 2018), have been tested in PDAC; hence, their usefulness is, in light of our current knowledge, unknown.

It is important to point out that K_Ca_3.1 activators are also targeting cancer cells and stromal cells. This has been shown for multiple cancer entities (see Mohr et al., (2019) for review) including PDAC (Jäger et al., 2004; Bonito et al., 2016; Storck et al., 2017). Riluzole and SKA-31 were successfully used to suppress the expansion of colorectal cancer cell lines, in combination with the K_V_11.1 channel blocker E4031 (Pillozzi et al., 2018) (see below for a detailed discussion of the challenges associated with targeting K_V_11 channels in PDAC therapy). Another study by Sun et al. showed that riluzole is capable of inhibiting the proliferation and partly killing several human PDAC cell lines (Sun et al., 2019). These effects, in combination with the aforementioned effect of K_Ca_3.1 activators in rebalancing T‐cell [K^+^]_i_, could synergistically act in the treatment of malignancies by targeting the K_Ca_3.1 channel.

Although riluzole has been defined and used as a K_Ca_3.1 activator (Pillozzi et al., 2018), this has to be viewed with caution since its specificity is very limited and it inhibits a series of other ion channels, such as K_V_11.1 (Pillozzi et al., 2018) and, most notably, several voltage-gated sodium channels (**Na_V_1.x**) (Song et al., 1997; Djamgoz and Onkal, 2012). Several human carcinomas express those Na_V_s, which promote invasiveness and metastasis under hypoxic conditions (Djamgoz et al., 2019). Inhibition of the hypoxia-sensitive persistent component of the Na_V_ current (INa_P_) by riluzole suppresses cancer cell invasiveness *in vitro* (Djamgoz and Onkal, 2012). There is evidence that aggressive PDAC cell lines express Na_V_1.5 and Na_V_1.6, but these channels do not seem to play a major functional role in PDAC cells. Na_V_ channel activity becomes measurable only upon EGFR inhibition (Bonito, 2017). Hence, we can suppose that riluzole will not influence PDAC growth by its direct action on Na_V_. The aforementioned effects of the “broad-spectrum” ion channel inhibitor riluzole on PDAC cell lines (Sun et al., 2019) are therefore more likely to be attributed to its action on the K_Ca_3.1 or K_V_11.1 channels. Nonetheless, the use of a broad-spectrum ion channel modulator can be justified when it yields the desired phenotypic effects (e.g., inhibition of migration and proliferation). Phenotypical drug screening is at least as successful as molecular screening which aims at a single molecularly defined target (Zheng et al., 2013).

Riluzole has pleiotropic effects, including dampening of excitotoxicity through interruption of glutamatergic transmission in the central nervous system (Benavides et al., 1985). This effect may be mediated by the inhibition of the activation of NMDA glutamate receptors (Debono et al., 1993). In addition to the inhibition of glutamatergic transmission, the beneficial effects of riluzole in the treatment of ALS may include inhibition of presynaptic voltage-gated Ca^2+^ channels and the inhibition of Na_V_ channels and block of the persistent Na^+^ currents in motoneurons ((Lamanauskas and Nistri, 2008), and see Cheah et al. (2010) for review). Thus, the use of riluzole as a K_Ca_3.1 activator may be limited by its effects on the central nervous system.

The action of Na_V_ inhibitors is further complicated as human immature dendritic cells express Na_V_1.7 (Zsiros et al., 2009). Silencing Na_V_1.7 shifts the membrane potential to more hyperpolarized values in CD1a^+^ immature dendritic cells. This results in decreased cell migration, which otherwise is a hallmark of a functionally immature dendritic cells (Kis-Toth et al., 2011). How this would impact in PDAC, a tumor known for its paucity in dendritic cell infiltration (Hegde et al., 2020), has not been explored yet.

The ATP-rich PDAC environment could also enhance immunosuppression through **P2X7**, which is present in both T lymphocytes (Feske et al., 2012) as well as in cancer (Giannuzzo et al., 2015) and stellate cells in PDAC (Haanes et al., 2012). This cation-selective ion channel is important for the infiltration of T lymphocytes into the tumor, since P2X7^−/−^ mice have a suboptimal immune response (De Marchi et al., 2019). Interestingly, inhibiting P2X7 with a selective antagonist like A740003 causes an increase in the percentage of infiltrating CD4^+^ cells and a significant drop in tumor weight and T_reg_ abundance (De Marchi et al., 2019). Whether these results, obtained with a mouse melanoma model, can be translated to immune modulation in a “cold” tumor like PDAC is unclear: the few reports about *in vivo* administration of P2X7 antagonists (AZ10606120 and A438079) did not analyze the involvement of the immune system (Mohammed et al., 2017) or used immunodeficient nude mice (Giannuzzo et al., 2016).

### Challenges of Using K_V_11 Channels as Therapeutic Target in PDAC

The most critical issue for using K_V_11.1 channel blockers in PDAC therapy have and still will be their cardiac side effects. Indeed, K_V_11.1 channels are highly expressed in the heart, representing the molecular correlate of the rapid repolarizing current I_Kr_ (Sanguinetti et al., 1995; Trudeau et al., 1995). Due to their peculiar gating properties, these channels are very effective in sustaining fast repolarizations in cardiac myocytes (Sanguinetti, 2010). For these reasons, pharmacological inhibition or malfunction of the K_V_11.1 channel can lead to potentially life-threatening arrhythmias, such as the long QT syndrome (LQT) (Sanguinetti, 2010; Mitcheson and Arcangeli, 2014).

Many K_V_11.1 channel blockers like the methanesulfonanilide E4031, which belong to class III of antiarrhythmic drugs (Ågren et al., 2019), require an open channel to gain access to the channel pore. They block the K^+^ flow and hence can delay heart repolarization (Mitcheson and Arcangeli, 2014; Chen et al., 2016), thus lengthening the QT interval. The capacity of different compounds to block K_V_11.1 channels and induce QT prolongation (the so-called “QT liability”) differs among structurally diverse compounds (Sanguinetti, 2010; Authier et al., 2017) so that some drugs effectively block K_V_11.1 channels but do not cause arrhythmias. These drugs are addressed as “nontorsadogenic K_V_11.1 blockers.” These drugs may represent good candidates for anticancer therapy (Cernuda et al., 2019).

An example is represented by *R*-roscovitine, a cyclin-dependent kinase (CDK) inhibitor that, although blocking K_V_11.1 currents in an open channel manner, shows no use dependency. This suggests rapid block and unblock kinetics (Cernuda et al., 2019). Thus, R-roscovitine could be used to target K_V_11.1 for PDAC therapy. Besides this compound, other studies have pointed out a potential for many “nontorsadogenic” K_V_11.1 blockers (Pointer et al., 2017) and drugs which bind the K_V_11.1 channel in a specific conformation (Becchetti et al., 2019). For example, the macrolide antibiotic clarithromycin, which is commonly used for bacterial infection, such as *H. pylori*, binds to K_V_11.1 in the closed conformation and shows a good antitumor activity in colorectal cancer (Petroni et al., 2020). Because it is already in clinical use for bacterial infections, it could also potentially serve as an antitumor drug in epithelial cancers like colorectal cancer and PDAC, as already shown in lymphomas (Ferreri et al., 2018).

Another approach is to target the K_V_11.1 subunit trafficking to the membrane. Up to now, several molecules have been shown to interfere with trafficking without having any effect on the channel itself. These include arsenic trioxide, geldanamycin, pentamidine, and probucol (Chen et al., 2016). Notably, arsenic trioxide has been and is still used as an anticancer drug, especially in leukemias (Hoonjan et al., 2018).

Yet another approach is to target K_V_11.1 channels with antibodies. The clinical potential of monoclonal antibodies has emerged in the last decade. However, due to the cardiovascular side effects seen for many drugs, targeting the K_V_11.1 channel with single-chain function blocking antibodies is also challenging and not preferred. K_V_11.1 channel–specific antibodies, such as the single-chain variable fragment (scFv), anti-Kv11.1 ScFv, are more relevant for cancer diagnosis, rather than for cancer treatment (Duranti et al., 2018). The risk of cardiac side effects can be reduced by employing bispecific antibodies. K_V_11.1 interacts with β1 integrins in PDAC, which is a tumor-specific feature of K_V_11.1 channels (Arcangeli and Becchetti, 2017). A convenient approach would therefore be to target this complex with a bifunctional single-chain diabody (scDb). This would increase the antibody–cancer cell specificity and reduce cardiovascular side effects. It may be taken as a proof of principle for therapeutic ion channel targeting that a monoclonal antibody against K_V_10.1 reduces tumor volume in a human pancreatic cancer xenograft mice model (Gómez-Varela et al., 2007).

Finally, antibody–drug conjugates (ADC) are biopharmaceutical drugs that are used for targeted therapy (e.g., Pahl et al. (2018)). The combination of cancer specificity and cytotoxicity makes them good candidates for next-generation cancer therapy (Parslow et al., 2016). An anti-K_V_11.1/β1 scDb could potentially be linked to cytotoxic drugs to create highly specific ADCs for pancreatic cancer treatment.

## Outlook

It remains an unsolved challenge to provide PDAC patients with an efficient therapy. New therapeutic strategies are urgently needed since none of the current treatment options yields satisfactory results. Presently, it is controversially debated whether the desmoplastic PDAC stroma and microenvironment can be targeted therapeutically. The conflicting results of these studies indicate that the properties and function of stromal cells such as pancreatic stellate cells and PDAC-infiltrating immune cells are far from being fully understood. In our view, an important class of membrane proteins has been neglected in this context: ion channels. They have been shown to be important drivers of aggressive cancer cell properties and related to all hallmarks of cancer. Being membrane proteins, ion channels are in a position to modulate, sense, and transduce properties of the tumor microenvironment. However, their roles in pancreatic cancer cells and in cells of the PDAC microenvironment are largely unexplored.

**FIGURE 3:**
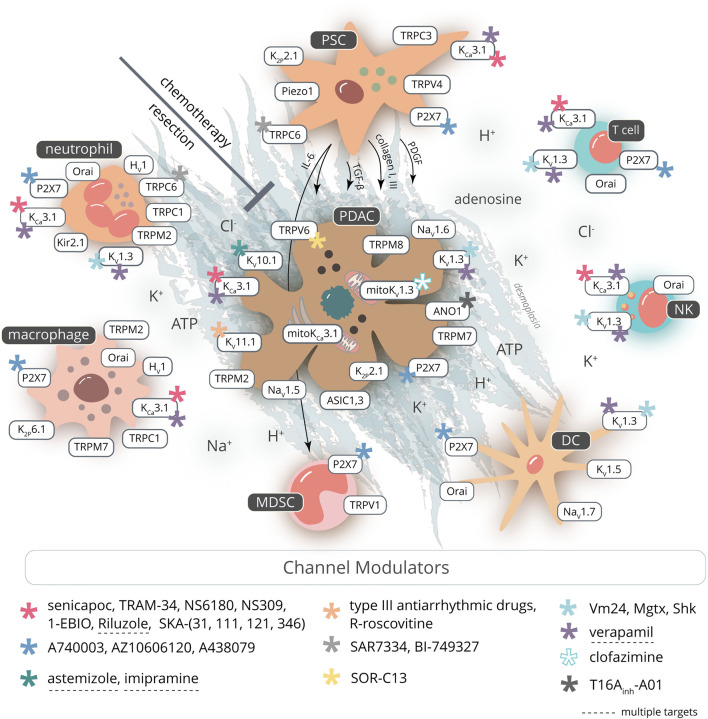
Ion channels in pancreatic ductal adenocarcinoma microenvironment and examples of channel modulators. Up to date, resection and chemotherapy are the only therapeutic approaches against PDAC. However, the fibrotic and acidic immunosuppressive tumor milieu hinders drug delivery and disturbs immune response. Ion channels expressed in tumor, stromal, and immune cells control cellular responses and therefore stand for putative drug targets. Here, we display those channels whose function has been investigated in cells of the PDAC tumor tissue. The respective modulators are indicated by asterisks.

We collected the current knowledge of ion channels in PDAC with a particular focus on immune and stromal cells and discussed the feasibility of therapeutic ion channel targeting. Figure 3 gives a graphical overview. Many tumors (mis-)use ion channels as highlighted for PDAC in this review. Based on the rapidly growing mechanistic knowledge about ion channels in PDAC, we are convinced that ion channel targeting offers a chance for therapeutic intervention.

We conclude that the choice of the right blocker or activator must be accurately rationalized, at least until we obtain enough data from electrophysiological measurements. Such data are still lacking for PDAC-infiltrating immune cells (Panyi et al., 2014). Similarly, there is hardly any electrophysiological data on stromal cells like PSCs. Studying ion channel activity directly in tumor-derived immune or stromal cells is an important issue. Their channel expression could show distinct differences from that of peripheral blood mononuclear cells or stromal cells isolated from a healthy pancreas. Moreover, it is imperative to consider the microenvironmental conditions under these circumstances. Up-regulation of channel expression may well be counterbalanced by characteristic properties of the tumor microenvironment such as the tumor acidity (Pethő et al., 2020). Since no K_V_1.3 activators are available at the moment (Chandy and Norton, 2016), K_Ca_3.1 activators appear promising in boosting tumor-infiltrating lymphocytes. Repurposing of riluzole is a good candidate for therapeutic K_Ca_3.1 activation; however, the lack of selectivity for K_Ca_3.1 might limit its application. At the same time, K_Ca_3.1 inhibitors could activate NK cells and stop cancer cells or stromal cells from proliferating and/or migrating. Only *in vivo* studies will reveal in the future which arm of the balance we should put our weights on when developing channel-targeting PDAC therapies. Thus, crossing K_Ca_3.1^−/−^ mice with transgenic breast cancer mouse models provided evidence that the impact K_Ca_3.1 targeting in cancer cells may be further modulated by K_Ca_3.1 targeting in noncancerous cells (Steudel et al., 2017). In this context, the right choice of the animal model is crucial, since mouse lymphocytes possess a different set of ion channels than rat and human ones (Beeton and Chandy, 2005). Such studies also need to evaluate repurposed drugs that are already in clinical use or have been tested in phase III clinical trials for other indications such as the K_Ca_3.1 blocker senicapoc (Ataga et al., 2011).

Another channel modulator that might serve as a target for drug repurposing is clofazimine. It inhibits K_V_1.3, thereby inducing apoptosis in PDAC cells and reducing primary tumor weight *in vivo* (Zaccagnino et al., 2017). It is already used for treatment of autoimmune disease and leprosy (Garrelts, 1991). However, K_V_1.3 block, inhibition of Na^+^/K^+^-ATPase of activated T‐cells (Anderson et al., 1986), and the release of ROS and PGE_2_ from bystander neutrophils (Anderson et al., 1986) inhibit T-cell proliferation and thus may compromise antitumor immunity as well. Follow-up studies are needed to tackle already existing ion channel modulators (inhibitors or activators) for a fast translation into the clinic for PDAC patients. We anticipate that combining innovative animal models with repurposed ion channel targeting drugs—potentially in combination with existing chemotherapeutic therapies—will open up exciting new options. Clearly, such studies would enormously profit from building larger (inter-)national research consortia that can address the diverse channel-related aspects of PDAC pathophysiology in a concerted manner.
